# Oligodendroglial Argonaute protein Ago2 associates with molecules of the *Mbp* mRNA localization machinery and is a downstream target of Fyn kinase

**DOI:** 10.3389/fncel.2015.00328

**Published:** 2015-08-25

**Authors:** Christina Müller, Isabelle Schäfer, Heiko J. Luhmann, Robin White

**Affiliations:** Institute of Physiology, University Medical Center of the Johannes Gutenberg University, MainzGermany

**Keywords:** oligodendrocyte, MBP, Argonaute, Fyn, non-coding RNA, mRNA localization, sncRNA715

## Abstract

Oligodendrocytes myelinate neuronal axons in the central nervous system (CNS) facilitating rapid transmission of action potentials by saltatory conduction. Myelin basic protein (MBP) is an essential component of myelin and its absence results in severe hypomyelination in the CNS of rodents. *Mbp* mRNA is not translated immediately after exit from the nucleus in the cytoplasm, but is transported to the plasma membrane in RNA transport granules in a translationally silenced state. We have previously identified the small non-coding RNA 715 (sncRNA715) as an inhibitor of *Mbp* translation associated with RNA granules. Argonaute (Ago) proteins and small RNAs form the minimal core of the RNA induced silencing complex and together recognize target mRNAs to be translationally inhibited or degraded. Recently, tyrosine phosphorylation of Ago2 was reported to be a regulator of small RNA binding. The oligodendroglial non-receptor tyrosine kinase Fyn is activated by neuronal signals and stimulates the translation of *Mbp* mRNA at the axon-glial contact site. Here we analyzed the expression of Ago proteins in oligodendrocytes, if they associate with *Mbp* mRNA transport granules and are tyrosine phosphorylated by Fyn. We show that all Ago proteins (Ago1-4) are expressed by oligodendrocytes and that Ago2 colocalizes with hnRNP A2 in granular cytoplasmic structures. Ago2 associates with hnRNP A2, *Mbp* mRNA, sncRNA715 and Fyn kinase and is tyrosine phosphorylated in response to Fyn activity. Our findings suggest an involvement of Ago2 in the translational regulation of *Mbp*. The identification of Ago proteins as Fyn targets will foster further research to understand in more molecular detail how Fyn activity regulates *Mbp* translation.

## Introduction

Fast and energy-efficient processing of information is the key task of the nervous system. In vertebrates this is accomplished by saltatory conduction of action potentials in myelinated neuronal axons (Nave and Werner, 2014). In the central nervous system (CNS) myelin is synthesized by oligodendrocytes which enwrap multiple axonal segments with plasma membrane, ultimately leading to a multilamellar compact myelin sheath (Snaidero et al., 2014; White and Krämer-Albers, 2014). Myelin basic protein (MBP) is the second most abundant myelin protein after PLP (proteolipid protein; de Monasterio-Schrader et al., 2012) and is required for myelin membrane compaction. Its interaction with the cytoplasmic leaflet of the oligodendroglial membrane seems to induce its polymerization and leads to the formation of a sieve-like structure defining the specific composition of lipids and proteins in myelin (Bakhti et al., 2014). The absence of functional MBP in rodents leads to severe hypomyelination in the CNS as demonstrated by the naturally occurring *shiverer* mouse or long evans *shaker* rat (Readhead and Hood, 1990; Kwiecien et al., 1998).

Interestingly, *Mbp* is transported from the nucleus to the plasma membrane as an mRNA and is translated locally at the axon-glial contact site (Müller et al., 2013). Presumably, this mechanism has evolved to prevent compaction of intracellular membranes by the basic protein product during transport which would impair cellular integrity. The localization of *Mbp* and other mRNAs takes place within the cell in ribonucleoprotein complexes referred to as RNA granules. The RNA binding protein hnRNP (heterogeneous nuclear ribonucleoprotein) A2 plays a key role as a trans-acting factor during transport. It binds to a specific sequence in the 3′ UTR of *Mbp* mRNA in the nucleus and mediates transfer to the cytoplasm and subsequently toward the plasma membrane on the microtubule network (Ainger et al., 1993; Carson et al., 1997; Hoek et al., 1998; Munro et al., 1999). Four splice variants (hnRNP A2, A2b, B1, and B1b) of the hnRNP A2/B1 gene have been reported which differ by the presence or absence of exons two and nine (Han et al., 2010). The activation of the oligodendroglial non-receptor tyrosine kinase Fyn by neuronal signals induces the phosphorylation of RNA granule-associated proteins such as hnRNP A2 and hnRNP F leading to *Mbp* translation at the axon-glial contact site (White et al., 2008, 2012; Kramer-Albers and White, 2011; Laursen et al., 2011; Wake et al., 2011).

It was unclear for a long time how *Mbp* mRNA is kept in a translationally silenced state during intracellular transport and development. We recently identified the oligodendroglial small non-coding RNA (sncRNA) 715 as an inhibitor of MBP synthesis which is associated with *Mbp* mRNA transport granules (Bauer et al., 2012). This 21 nucleotide long RNA was recently suggested to be a small rDNA-derived RNA (srRNA) and may originate from the 5′ externally transcribed spacer (ETS) sequence of 45S pre-ribosomal RNA (Wei et al., 2013). Chronic demyelinated multiple sclerosis lesions contain oligodendrocyte precursor cells (OPCs) with *Mbp* mRNA but no MBP protein. In these lesions the levels of sncRNA715 are significantly increased and may block *Mbp* translation (Bauer et al., 2012). Abnormally high levels of sncRNA715 in these MS lesions could be one of the reasons why resident OPCs fail to differentiate and remyelinate neuronal axons coinciding with clinical decline in patients.

The precise molecular events of sncRNA715-mediated inhibition of *Mbp* mRNA translation have not been unraveled so far. SncRNAs such as endogenous siRNAs or miRNAs require Argonaute (Ago) proteins to block the translation of targeted mRNAs and srRNAs are at least associated with Ago proteins (Wei et al., 2013). Four Ago proteins (Ago1-4) have been identified in vertebrates and sncRNA-Ago complexes are the core unit of the RNA-induced silencing complex (RISC) which mediates target mRNA degradation or translational repression depending on the sequence complementarity of the sncRNA and the target mRNA (Cenik and Zamore, 2011). It was previously shown that Ago proteins are phosphorylated on Y393 and Y529 and that Y529 phosphorylation affects miRNA binding while phosphorylation of Y393 influences interaction of Ago2 with the miRNA processing enzyme Dicer (Rudel et al., 2011; Shen et al., 2013; Yang et al., 2014).

In order to obtain a better understanding of the molecular components regulating the repression and stimulation of *Mbp* mRNA translation we analyzed a potential involvement of Ago proteins in oligodendrocytes. We found that Ago1-4 are expressed by primary oligodendrocytes and by the immortalized OPC line Oli-*neu*. Ago2 co-immunoprecipitates with hnRNP A2 and colocalizes with hnRNP A2 in primary oligodendrocytes. Moreover, *Mbp* mRNA and sncRNA715 co-immunoprecipitate with Ago2. We also found that Ago2 is a downstream target of Fyn kinase and both molecules co-distribute in oligodendroglial processes.

In summary we identified Ago2 as a novel Fyn target which is associated with the *Mbp* mRNA localization pathway in oligodendrocytes.

## Materials and Methods

### Plasmids, Antibodies, 715-Mimic

Generation of the wildtype (WT) Fyn and constitutive active Fyn (Fyn+) constructs were described earlier (White et al., 2008). FLAG/HA-Ago constructs were purchased from Addgene (pIRESneo-FLAG/HA Ago1 plasmid 10820, pIRESneo-FLAG/HA Ago2 plasmid 10821, pIRESneo-FLAG/HA Ago3 plasmid 10823, pIRESneo-FLAG/HA Ago4 plasmid 10824; Meister et al., 2004). Cloning of the FLAG-A2b vector (pcDNA3.1 backbone) was performed using standard molecular cloning techniques using EcoRI and XhoI restriction and the following primers in the PCR on Oli-*neu* derived cDNA, 5′-CGAATTCATGGAGAGAGAAAAGG-3′ and 5′-GGCTCGAGT TAATATCTGCTCCTTCCACC-3′. MBP14-3′UTR plasmid was kindly provided by M. Simons, Göttingen. The hnRNP A2-Myc/His construct was generated by cloning the hnRNP A2 ORF lacking the STOP codon into the EcoRI/XhoI sites of the pcDNA-TO-Myc-His backbone vector (Life Technologies) using the following primers in the PCR reaction on Oli-*neu* derived cDNA, 5′-CGAATTCATGGAGAGAGAAAAGG-3′ and 5′-GGCTCGAGATATCTGCTCCTTCCACC-3′.

Monoclonal antibodies were used against Ago isoform 2 (mouse, abnova) 1:500 in WB and 1:50 in ICC, phosphotyrosines pTyr (clone 4G10, mouse, Merck Millipore) 1:1000 in WB, MBP (rat, Serotec) 1:500 in WB and 1:50 in ICC, HA (rat, Roche Applied Science) 1:1000 in WB, hnRNP A2 (mouse, Sigma–Aldrich) 1:1000 in WB, hnRNP A2 EF67 (mouse, W. Rigby, Hanover) 22.5 μg in IP with 50 μl dynabeads, Myc (mouse 9E10, C. Pietrzik, Mainz) 1:50 in ICC, α-Tubulin (mouse, Sigma–Aldrich) 1:5000 in WB, FLAG-M2 (mouse, Sigma–Aldrich) 1:1000 in WB.

Polyclonal antibodies were used against Ago isoform 1 and 2 (rabbit, Cell signaling) 1:1000 in WB, GAPDH (rabbit, Bethyl) 1:5000 in WB, hnRNP A2 (rabbit, Sigma–Aldrich) 1:50 in ICC, beta-Actin (rabbit, Sigma–Aldrich) 1:1000 in WB, Fyn kinase (rabbit, Santa Cruz) 1:50 in ICC and active Fyn kinase (Src418; rabbit, Life Technologies) 1:1000 in WB.

Secondary antibodies were used from Dianova (HRP-coupled and Cy-dyes), from Life Technologies (Alexa- Dyes) or from Bethyl (DyLight-dyes).

Synthetic sncRNA 715 was purchased from Qiagen (715-mimic; 5′-C-UCCGUGCACACCCCCGCGUG-3′).

### Cell Culture and Transfections

Oli-*neu* cells (kindly provided by J. Trotter, Mainz) were cultured as described before (White et al., 2012). Primary mouse OPCs were established from C57BL/6 mice postnatal day 9 using the Neural Tissue Dissociation Kit with Papain (Miltenyi Biotec) and Anti-AN2 Microbeads (Miltenyi Biotec) according to manufacturer’s protocol and cultured in MACS Neuro Medium containing 1% Pen/Strep, 1% L-Glutamine and 2% NeuroBrew. Primary cultures were grown in poly-L-lysine-coated (PLL) culture dishes.

HEK293T cells were cultured in DMEM + Glutamax (Life Technologies) supplemented with 10% fetal calf serum and 10000U Penicillin/Streptomycin (Life Technologies).

Oli-*neu* cells and HEK293T cells were transfected using Fugene HD (Promega). Plasmid-DNA and Fugene HD were mixed in a 1:1.5 ratio. Per 10 cm culture dish 500 μl DMEM + Glutamax were mixed with 5 μg Plasmid-DNA and 7.5 μl Fugene HD vortexed and incubated for 15 min at room temperature before application to the cells. For co-transfection 2.5 μg of each Plasmid-DNA was used. After about 36 h post-transfection cells were lysed and analyzed by Western blotting.

To increase endogenous sncRNA715 levels in Oli-*neu*, cells were transfected with 80 pmol synthetic sncRNA715 (715-mimic) using Lipofectamine RNAiMAX Transfection Reagent according to the manufacturer’s protocol (Life Technologies).

Entire murine optic nerves were extracted after decapitation from mice at postnatal day 1 (P1) or 10 (P10). The nerves were dissociated using a TissueRuptor (Qiagen) in lysis buffer (50 mM Tris, 150 mM NaCl, 1 mM EDTA, 1% Triton X-100) containing protease and phosphatase inhibitors (Complete Mini EDTAfree and PhosStop, both Roche Applied Science). After 45 min incubation at 4°C under permanent rotation, nuclei were removed by centrifugation at 2000 × *g* for 5 min and 4°C. The supernatants were analyzed by Western blotting.

### Ethics Statement

Experiments were performed in accordance with the animal policies of the University of Mainz, approved by the German Federal State of Rhineland-Palatinate, in accordance with the European Community Council Directive of November 24, 1986 (86_609_EEC). Great care was taken to prevent the animals from suffering. Rats and mice were sacrificed by decapitation after isoflurane anesthesia. The experiments were performed under the German animal welfare law (§4) by persons with specific knowledge and skills. All animals were killed for scientific purposes before using organ material for these studies. A special approval for killing for scientific purposes under §4 of the animal welfare law is not necessary.

### Immunfluorescence and Microscopy

Primary oligodendrocytes were cultured on PLL-coated coverslips. At specific timepoints cells were fixed for 15 min at room temperature in 4% (w/v) paraformaldehyde and permeabilized with 0.1% (v/v) Triton X-100 in PBS for 2 min. After blocking with 10% horse serum in DMEM 15 min at room temperature primary antibodies were applied over night at 4°C. To visualize proteins secondary antibodies conjugated with DyLight488 (1:100), Alexa568 (1:400 – 1:600) or Cy5 (1:300) were used in blocking medium for 30 min at room temperature. Nuclei were stained with 4′,6-diamidino-2-phenylindole (DAPI, 1:7500) for 5 min at room temperature. Stained cells were mounted in Moviol.

Images were acquired using a TCS SP5 Confocal Microscope (Leica) with a HCX PL APO CS 63x/1.4 oil UV objective connected to a fast resonance scanner. Images were edited using the software LAS AF Version 2.6.3 (Leica Microsystems CMS GmbH), FIJI software (Schindelin et al., 2012), Adobe Photoshop CS4 and Adobe Illustrator CS4.

### Quantification of Colocalization using Mander’s Coefficient

The extent of colocalization of Ago2 with either hnRNP A2 or Fyn kinase was measured quantitatively by Mander’s correlation coefficient (MCC), Pearson’s correlation coefficient (PCC) and Cost’s test using Fiji software with the JACoP plugin as described previously (Manders et al., 1993; Bolte and Cordelieres, 2006). Regions of interest in the area of oligodendroglial processes were selected using the rectangular selection tool to analyze colocalization in the periphery of oligodendrocytes. Two different Mander’s coefficient values are given (M1 and M2), which describe the independent contributions of two selected channels to the pixels of interest. M1 represents the fraction of the red channel (Ago2) in regions containing green signal (hnRNP A2 or Fyn kinase; **Supplementary Figures S2B** and **S3B**) and M2 accounts for the fraction of the green channel in regions containing red signals (**Supplementary Figures S2A** and **S3A**). Here we focused on the M2 values to show the relative colocalization of hnRNP A2 or Fyn with Ago2 compared to total Ago2. MCC values range from 0 to 1 while 0 means no colocalization (0%) and 1 perfect colocalization (100%). PCC values range from 0 to 1 and Cost’s test gives values for r (original), which should be the same as PCC, r (randomized) which is supposed to be near 0 when it is real colocalization and the *P*-value in %, where values higher than 95% mean significance of the colocalization.

### Cell Lysis and Western Blotting

Cells were washed with ice cold PBS and scraped off in lysis buffer (50 mM Tris, 150 mM NaCl, 1 mM EDTA, 1% Triton X-100) containing protease and phosphatase inhibitors (Complete Mini EDTAfree and PhosStop, both Roche Applied Science). The lysate was incubated on a rotating wheel at 4°C for 45 min and afterward cleared from nuclei and debris by centrifugation at 2000 × *g* and 4°C for 5 min.

Separation of proteins was performed by SDS-PAGE using a Mini PROTEAN system (Bio-Rad) or Novex NuPAGE SDS-PAGE Gel system (Life Technologies) and transferred onto Roti-PVDF membranes (0.45 μm, Roth) using a Mini TransBlot Electrophoretic Transfer Cell device (Bio-Rad). Precision Plus Protein Standard (Bio-Rad) was used as a marker. Membranes were blocked with 4% (w/v) milk (Roth) in TBST [50 mM Tris, 150 mM NaCl, pH 7.2, 0,1% (v/v) Tween 20] for 30 min at room temperature. Binding of primary antibodies was carried out either at 4°C over night or for 1 h at room temperature. Suitable secondary antibodies (coupled to horseradish peroxidase, Dianova) were incubated for 30 min at room temperature. All antibodies were diluted in blocking medium. Image acquisition was performed in a ChemiDoc XRS system using Imagelab software (Biorad).

### Immunoprecipitation, His Isolation

Immunoprecipitations (IP) of tyrosine phosphorylated proteins were performed with anti-phosphotyrosine agarose conjugated beads (clone 4G10, Millipore). Beads were washed once with lysis buffer and incubated with cell lysates at 4°C over night at permanent rotation. After incubation, protein-bead complexes were washed three times with ice cold PBS and bound proteins were eluted with 1x LDS Sample buffer for 10 min at 70°C.

Immunoprecipitations of FLAG-tagged proteins was carried out by using anti-FLAG M2 Magnetic beads (Sigma–Aldrich). FLAG-beads were washed once with TBST and incubated with the appropriate cell lysate for 2 h at 4°C on a rotation wheel. After washing three times with lysis buffer, proteins were eluted with 13.5 μg FLAG-Peptide (Sigma–Aldrich) in 30 μl PBS with Phosphatase- and Protease Inhibitors (Complete Mini EDTAfree and PhosStop).

For Isolation of His-tagged proteins, Dynabeads His Tag Isolation and Pulldown magnetic beads were used (Life technologies). 50 μl bead slurry per reaction was washed twice with wash buffer 1 [300 mM NaCl, 50 mM Tris, 150 mM Imidazol, Protease and Phosphatase Inhibitors (Roche Applied Science)]. Cells were lysed in lysis buffer (50 mM Tris, 300 mM NaCl, 150 mM imidazole, 1% (v/v) Triton X100, Protease, and Phosphatase Inhibitors) as described above, mixed 1:1 with wash buffer 1 and incubated for 30 min at RT at permanent rotation. The supernatant was discarded and bead complexes were washed thrice for 10 min at RT with wash buffer 2 (50 mM Tris, 300 mM NaCl, 500 mM imidazole, protease, and phosphatase inhibitors) on a rotation wheel. One last washing step was performed in wash buffer 3 (50 mM sodiumphosphate pH 8.0, 300 mM NaCl, 0,01% (v/v) Tween 20, 2M Imidazol, Protease, and Phosphatase Inhibitors). Proteins were eluted by adding 1x LDS sample buffer and heating at 70°C for 10 min. All IPs were further analyzed by Western blotting.

### RNA Extraction, Reverse Transcription and PCR

Total RNA of Oli-*neu* cells and primary OPCs were extracted using either the RNeasy Mini Kit or miRNeasy Mini Kit (both Qiagen). Reverse transcription of mRNAs was performed with the Transcriptor High Fidelity Reverse Transcription Kit (Roche Applied Science). SncRNA715 was reverse transcribed by the TaqMan MicroRNA Reverse Transcription Kit with stem-loop RT primers specific for sncRNA715 sequence (Applied Biosystems) and amplified with the Taqman Universal Master Mix (Roche Applied Science) with specific primers and probes for sncRNA715 (Applied Biosystems). RT-PCR primers specific for rat MBP: 5′-AACATTGTGACACCTCGAACA-3′ and 5′-TGTCTCTTCCTCCCCAGCTA-3′. PCR of Ago1-4 mRNAs was carried out using the Pfu-DNA Polymerase according to manufacturer’s protocol. Primers were specifically designed to differentiate the four different murine Ago proteins (Ago1: 5′-AGCGGCAGGTGCCTAC-3′ and 5′-GGATCTGGCCACTGACC3-′, Ago2: 5′-ATATGCC TTCAAACCTCCACC-3′ and 5′-CCAGGGCCTGGATCGTC-3′, Ago3: 5′-ACAAGCCTGTCAGCACTAAC-3′ and 5′-CAGAC TTCTAGTGGCAGGTATG-3′, Ago4: 5′-GGAGCTCCTCTACA GCCAGG-3′ and 5′-CAAGCCGGGCATAATATGC-3′. Ago1-4 PCR products and 100 bp DNA Step Ladder (Promega) were separated on 1% agarose gels and stained with ethidium bromide (EtBr). RT-PCR products of *Mbp* mRNA and sncRNA715 were separated on 4% agarose gels and stained with EtBr.

## Results

### Ago Expression in Oligodendrocytes

**FIGURE 1:**
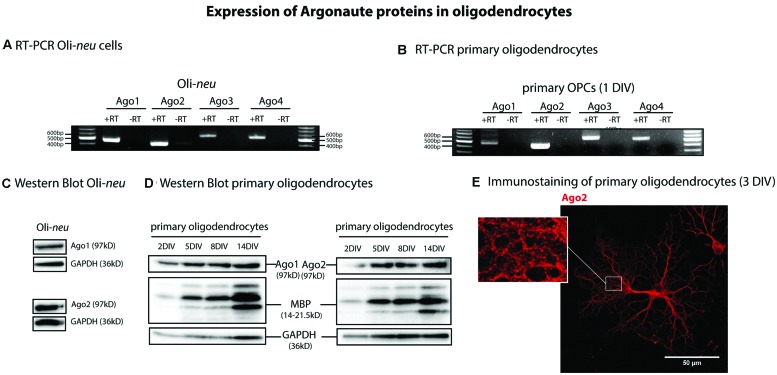
**Argonaute expression in oligodendrocytes. (A,B)** Reverse transcription and PCR (RT-PCR) on total RNA from Oli-*neu* **(A)** and primary mouse oligodendrocyte precursor cells (OPCs) cultured 1 day *in vitro* (OPC, 1DIV) **(B)** using specific primers for Ago1, 2, 3, 4 (+RT). -RT indicates a control reaction lacking reverse transcriptase enzyme to exclude contamination by genomic DNA. PCR products were visualized in ethidium bromide-stained 1% agarose gels. **(C,D)** Detection of Ago1 and Ago2 in Oli-*neu* cells **(C)** and in differentiating primary mouse oligodendrocytes **(D)** by Western blots at the indicated time points. Myelin basic protein (MBP) levels were analyzed to assess differentiation status of primary oligodendrocytes and GAPDH levels were used as a loading control. **(E)** Ago2 immunostaining of primary mouse oligodendrocytes cultured 3DIV. One single confocal plane is shown. Bar, 50 μm.

Four Ago proteins (Ago1-4) have been identified in vertebrate tissues (Pfaff and Meister, 2013). In order to find out which of these Ago proteins are expressed by oligodendrocytes, we performed RT- PCRs with specific primers for Ago1, 2, 3, and 4. We analyzed mRNA isolated from the OPC line Oli-*neu* as well as from cultured primary murine OPCs. We found that mRNA for Ago1-4 is expressed in the Oli-*neu* cell line and by primary cells (**Figures 1A,B**). We confirmed the expression of Ago1 and 2 in Oli-*neu* cells (**Figure 1C**) and differentiating cultured primary oligodendrocytes (**Figure 1D**) and in the optic nerve of mice postnatal day 1 (P1) and 10 (P10; **Supplementary Figure S1**) on protein levels by SDS-PAGE and Western blotting. In these experiments glyceraldehyde 3-phosphate dehydrogenase (GAPDH) served as a loading control and in primary cells and in the optic nerves the increasing amounts of MBP confirm the increasing degree of oligodendroglial differentiation over time in culture or *in vivo* (2DIV-14DIV, P1 and P10). Ago1 and Ago2 proteins are expressed during all stages of differentiation in cultured oligodendrocytes as well as *in vivo*. Immunostainings and confocal imaging visualizes Ago2 in the cell bodies and processes of MBP expressing, differentiating oligodendrocytes cultured 3DIV (**Figure 1E**). Interestingly, the Ago2 staining appears in a granular pattern especially in developing membrane sheet structures (see magnification in **Figure 1E**). We focused our subsequent experiments on Ago2.

### Ago2 is Associated with the *Mbp* mRNA Transport Pathway

In oligodendrocytes *Mbp* mRNA is transported through the oligodendroglial cytoplasm toward the periphery in RNA granules which largely depends on the *Mbp* mRNA binding protein hnRNP A2 (Müller et al., 2013). Due to a similar granular staining pattern of Ago2 in our experiments, we analyzed if Ago2 is associated with the *Mbp* mRNA transport pathway.

**FIGURE 2:**
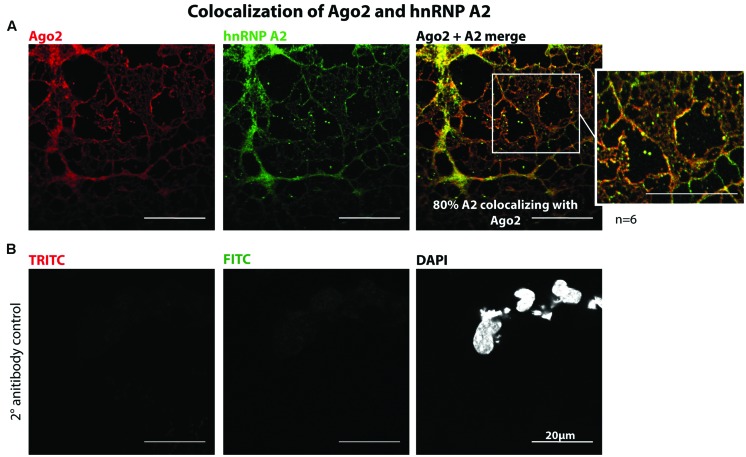
**Colocalization of Ago2 and hnRNP A2 in oligodendrocytes. (A)** Mouse primary oligodendrocytes were fixed at 5DIV and immunostained for Ago2 and hnRNP A2. One single confocal plane is shown. Colocalization was quantified using ImageJ with JACoP plugin and Mander’s coefficient M2. 6 independent experiments (*n* = 6) show a mean colocalization of hnRNP A2 with Ago2 of 80.18% ± 8.3% (for details see **Supplementary Figure S2**). Bars, 20 μm. **(B)** Secondary antibody control of immunostaining shown in A to exclude unspecific secondary antibody signals. The coupled dyes are indicated. One single confocal plane is shown. Bars, 20 μm.

We co-immunostained primary oligodendrocytes with Ago2 and hnRNP A2 antibodies which recognizes all hnRNP A2/B1 isoforms and determined the MCC M2 revealing approximately 80% colocalization in cellular processes and at their distal edges (**Figure 2A**, *n* = 6, for quantification see **Supplementary Figure S2**). In addition we estimated the PCC and checked for significance of the measured colocalization with Cost’s tests which confirmed the results of the quantification performed with MCC (**Supplementary Figure S2B**). We used MCC instead of PCC for analysis because it is stated to be more suitable for fluorescent signals distributed to different kinds of compartements and MCC is independent of signal intensities (Dunn et al., 2011; Emi et al., 2012). Taken together we could reveal a significant colocalization of Ago2 and hnRNP A2. We used only the M2 value to relate the colocalization of hnRNP A2 with Ago2 to total Ago2, to obtain the percentage of hnRNP A2 associated with Ago2, because not all hnRNP A2 proteins are associated with *Mbp* mRNA transport granules and therefore probably also not associate with Ago2. Only regions of interest in the periphery of oligodendrocytes were analyzed (**Supplementary Figure S2C**) where we expected the highest degree of colocalization. To demonstrate the specificity of the immunostainings, we included a secondary antibody control experiment in which the primary antibodies were omitted which only showed a faint, if any, background signal (**Figure 2B**).

**FIGURE 3:**
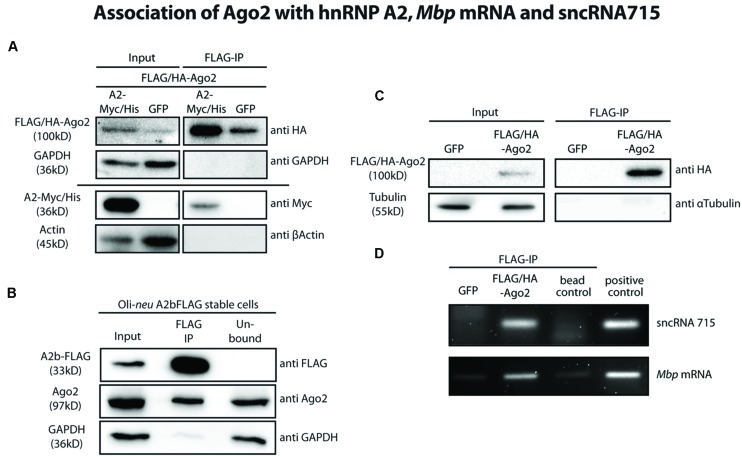
**Ago2 associates with the *Mbp* mRNA transport machinery. (A)** FLAG-tagged proteins were immunoprecipitated from FLAG/HA-Ago2 and A2-Myc/His or eGFP overexpressing Oli-*neu* cells using FLAG-M2 magnetic beads to analyze interaction of Ago2 and hnRNP A2 (lanes 3 and 4). The horizontal line separates two independent blots with equal sample loading. Lanes 1 and 2 show 2.5% of the protein input that was used for the immunoprecipitation (IP). Proteins were analyzed by western blotting using antibodies against HA- and Myc-tag. Antibodies against GAPDH and β-Actin were used to show specificity of the IP. **(B)** Immunoprecipitation of FLAG-tagged proteins from hnRNP A2b-FLAG stably expressing Oli-*neu* cells using FLAG-M2 magnetic beads (lane 2). Proteins were analyzed by Western blotting using antibodies against FLAG-tag and Ago2. Antibodies against GAPDH were used to show specificity of the IP. Lane 1 (input) shows 2.5% of the protein input that was used for the IP and lane 3 (unbound) the unbound protein fraction after incubation with the FLAG antibody beads. **(C)** IP of FLAG-tagged proteins from FLAG/HA-Ago2 or GFP overexpressing Oli-*neu* cells using FLAG-M2 magnetic beads (FLAG-IP, lanes 3 and 4). Lanes 1 and 2 (input) show 2.5% of the protein input that was used for the IP. Proteins were analyzed by western blotting using antibodies against HA-tag and α-Tubulin to show the specificity of the IP. **(D)** RNA was extracted from IP eluate of the experiment shown in C and analyzed by RT-PCR for sncRNA715 and *Mbp* mRNA. PCR products of *Mbp* (88 nt) and sncRNA715 (∼60 nt, due to the use of hairpin primers) were visualized in an ethidium bromide-stained 4% agarose gel. Positive control for sncRNA715 is the synthetic sncRNA715 (715-mimic) and for *Mbp* mRNA total RNA from primary rat oligodendrocytes at 14DIV. Negative control (bead control) reveals IP-reaction without addition of protein lysate to ensure specificity of the signals.

We then assessed a potential association of Ago2 proteins with the hnRNP A2- dependent *Mbp* mRNA transport machinery biochemically by co-immunoprecipitation experiments. We overexpressed FLAG/HA-tagged Ago2 and Myc/His-tagged hnRNP A2 or eGFP as a control in Oli-*neu* cells and immunoprecipitated FLAG/HA-Ago2. As shown in **Figure 3A**, hnRNP A2-Myc/His coimmunoprecipitated with FLAG/HA-Ago2. In these experiments, GAPDH and beta-Actin served as specificity controls for the IP and could not be detected in these.

It was postulated that isoform hnRNP A2b is predominantly localized in the cytoplasm (Han et al., 2010) and would thus be very likely to interact with cytoplasmic Ago proteins. We generated Oli-*neu* cells stably expressing a FLAG-tagged hnRNP A2b isoform. We then immunoprecipitated FLAG-hnRNP A2b from these cells using anti-FLAG M2 magnetic beads and could show by Western blotting that endogenous Ago2 coimmunoprecipitated with FLAG-hnRNP A2b (**Figure 3B**).

We next analyzed if sncRNA715 is associated with Ago2 in oligodendroglial cells. We transfected Oli-*neu* cells with plasmids coding for the most abundant rodent splice variant MBP14 including its 3′UTR and with eGFP (control) or FLAG/HA-Ago2 vectors in addition to synthetic sncRNA715 (715-mimic). We lysed the transfected cells and immunoprecipitated FLAG/HA-Ago2 using anti-FLAG M2 magnetic beads. The beads were then separated and processed further for protein and RNA analysis by Western blotting and RT-PCR, respectively. Western blots confirmed the immunoprecipitation of FLAG/HA-Ago2 (**Figure 3C**) and the association of sncRNA715 and *Mbp* mRNA in this fraction could be shown by RT-PCR as both RNAs are strongly enriched in the presence of FLAG/HA-Ago2 protein (**Figure 3D**).

Taken together the colocalization of Ago2 proteins with hnRNP A2 and the copurification of Ago2 with hnRNP A2, *Mbp* mRNA and sncRNA715 strongly suggests an involvement of Ago2 proteins in sncRNA715-mediated translational repression of *Mbp* mRNA in oligodendrocytes.

### Ago2 is a Downstream Target of Fyn Kinase

**FIGURE 4:**
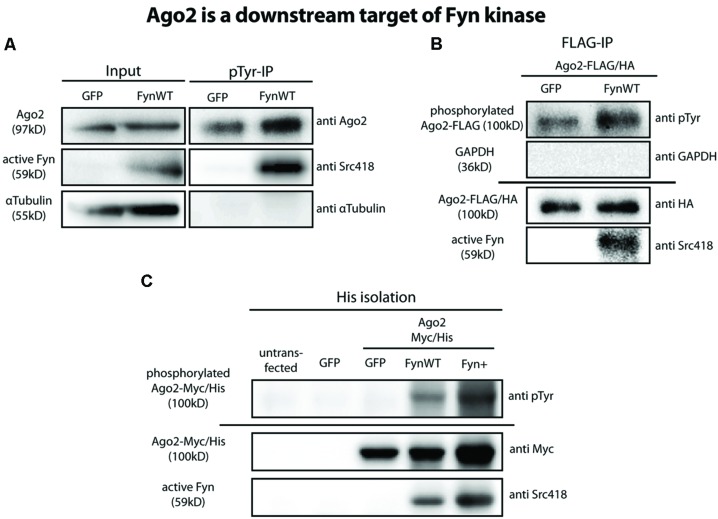
**Ago2 is a downstream target of Fyn. (A)** Tyrosine phosphorylated proteins were immunoprecipitated from GFP or FynWT overexpressing Oli-*neu* cells using the monoclonal anti-phospho-tyrosine antibody 4G10 (pTyr-IP, lanes 3 and 4). Lanes 1 and 2 (input) show 2.5% of the protein input that was used for the IP. Proteins were analyzed by Western blotting using antibodies against Ago2, active Fyn (SrcpY418) and α-Tubulin. **(B)** IP of FLAG-tagged proteins from FLAG/HA-Ago2 and GFP or FynWT overexpressing Oli-*neu* cells using FLAG-M2 magnetic beads. The horizontal line separates two independent blots with equal samples loading. Proteins were analyzed by Western blotting using antibodies against tyrosine-phosphorylated proteins (pTyr, clone 4G10), HA-tag, active Fyn (SrcpY418) and GAPDH. **(C)** His-Isolation of untransfected or eGFP or Ago2-Myc/His and eGFP or FynWT or Fyn+ overexpressing HEK293T cells. The horizontal line separates two independent blots with equal sample loading. Proteins were analyzed by Western blotting using antibodies against tyrosine-phosphorylated proteins (pTyr, clone4G10), Myc-tag and active Fyn (Src418).

The non-receptor tyrosine kinase Fyn has previously been identified as a regulator of oligodendroglial *Mbp* translation (Kramer-Albers and White, 2011; Müller et al., 2013). Moreover, the phosphorylation of Ago2 on tyrosine residue 529 (Y529) by a so far unknown tyrosine kinase was suggested to release bound miRNAs from Ago2 to regulate miRNA-mediated translational repression (Rudel et al., 2011). We hence analyzed a potential phosphorylation of Ago proteins by Fyn. We transfected Oli-*neu* cells with eGFP or WT Fyn (FynWT) plasmids and immunoprecipitated tyrosine-phosphorylated proteins. We then analyzed these samples by Western blotting with Ago2-specific antibodies and found a strong increase in the levels of tyrosine phosphorylated Ago2 after FynWT transfection (**Figure 4A**). We confirmed increased levels of active Fyn in the FynWT-transfected cells using the Phospho-Src pTyr418 antibody recognizing active Src family kinases including Fyn. Tyrosine-(auto) phosphorylated Fyn was also found in the phosphotyrosine immunoprecipitation as expected and the absence of α-tubulin served as a specificity control.

In an additional approach we co-transfected Oli-*neu* cells with plasmids coding for FLAG/HA-Ago2 and either eGFP or wild-type Fyn (FynWT). Using anti-FLAG M2 magnetic beads, we immunoprecipitated FLAG/HA-Ago2 from these cells after 2 days and used phospho-tyrosine specific antibodies to determine if the immunoprecipitated FLAG/HA-Ago2 is tyrosine-phosphorylated in the presence of active Fyn. As shown in **Figure 4B**, FLAG/HA-Ago2 is more strongly tyrosine-phosphorylated in FynWT-transfected cells compared to eGFP-transfected cells. Notably, active Fyn coimmunoprecipitates with FLAG/HA-Ago2 alluding to a direct interaction of active Fyn and Ago2. The absence of GAPDH in the immunoprecipitation served as a specificity control. We performed the same coexpression and immunoprecipitation experiments with FLAG/HA-Ago1, FLAG/HA-Ago3, and FLAG/HA-Ago4 plasmids instead of FLAG/HA-Ago2 and found that the other Ago proteins are tyrosine-phosphorylated in response to Fyn activity as well (data not shown).

To exclude a potential phosphorylation of the FLAG/HA-tag, which itself contains several tyrosine residues, in the recombinant FLAG/HA-Ago2 protein by Fyn, we generated a Myc- and His-tagged Ago2 plasmid (Ago2-Myc/His) lacking any tyrosines in the tag sequence. We co-transfected this construct into HEK293T cells with eGFP or FynWT or the constitutively active Fyn mutant Fyn+ (White et al., 2012) and subsequently purified the Ago2 proteins by their His-tag using cobalt beads. Similar to FLAG/HA-Ago2, Ago2-Myc/His is strongly phosphorylated in FynWT and Fyn+ transfected cells (**Figure 4C**). Moreover, active Fyn could be co-purified with Ago2-Myc/His.

**FIGURE 5:**
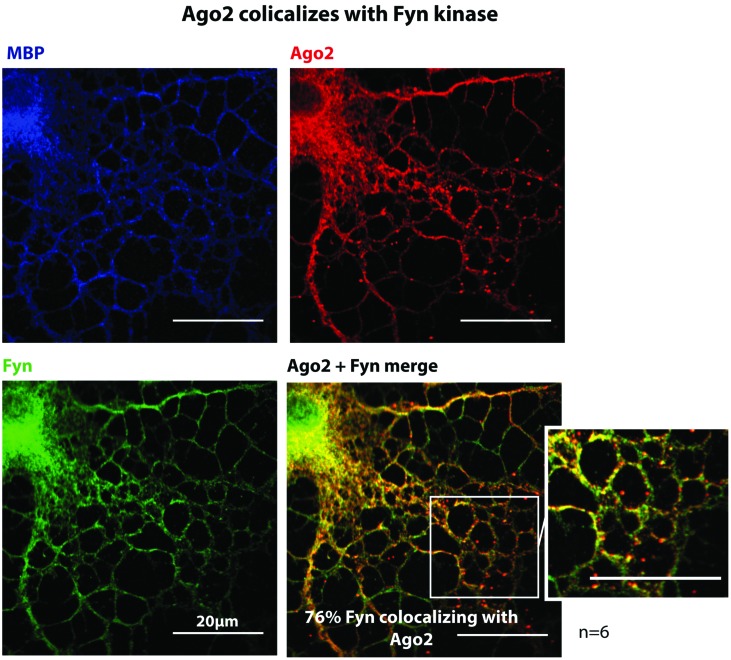
**Ago2 colocalizes with Fyn kinase.** Primary mouse oligodendrocytes 3DIV were fixed and stained for Ago2, Fyn-kinase and MBP. One single confocal plane is shown. Colocalization was quantified using ImageJ with JACoP plugin and Mander’s coefficient M2. 6 independet experiments (*n* = 6) show a mean colocalization of Fyn with Ago2 of 76.45% ± 4.8% (for details see **Supplementary Figure S3**). Bars, 20 μm.

Myelin basic protein expressing primary oligodendrocytes were co-immunostained with Fyn and Ago2 antibodies and show a co-distribution of Fyn and Ago2 in the processes and the cell body (**Figure 5**) further supporting a direct interaction as observed biochemically (**Figures 4B,C**). In order to quantify the degree of co-distribution in regions of interest in the cell periphery (**Supplementary Figure S3C**), where Fyn is active (White and Krämer-Albers, 2014) we determined the Mander’s colocalization coefficient M2 of Fyn and Ago2 in six individual experiments and found a mean 76% co-distribution (see **Supplementary Figure S3** for quantification details). PCC analysis and of Cost’s tests confirmed this value as significant co-distribution (**Supplementary Figure S3B**).

## Discussion

In eukaryotic cells, post-transcriptional control of gene expression guarantees tightly adjustable protein synthesis. sncRNAs can inhibit the translation of mRNAs and can thereby control the synthesis of proteins in a temporal- and also a spatial manner if the targeted mRNAs are localized. This implies the possibility of a cellular system to respond rapidly to environmental cues as stored mRNAs can be translated immediately, bypassing transcriptional events. During CNS myelination, oligodendrocytes synthesize large amounts of membrane proteins and lipids in order to increase their surface area required to enwrap neuronal axons. As the formed myelin sheath corresponds to axonal properties such as diameter and activity and a single oligodendrocyte may myelinate 50 different axonal segments simultaneously, an appropriate organization of protein and lipid synthesis and distribution is crucial (White and Krämer-Albers, 2014). MBP is the second most abundant protein in CNS myelin (de Monasterio-Schrader et al., 2012) and severe CNS hypomyelination in rodents lacking functional MBP (Readhead and Hood, 1990; Kwiecien et al., 1998) demonstrates that MBP synthesis is required to form compact myelin. As the lack of other myelin proteins does not result in such striking hypomyelinated phenotypes, MBP has been denoted the “executive molecule of myelin” (Boggs, 2006). The translation of *Mbp* mRNA occurs in response to neuronal triggers locally at the axon-glial contact site (Müller et al., 2013). Deciphering the molecular pathways controlling the synthesis of MBP is required to understand the initiation of myelin formation.

### Ago2 and *Mbp* Localization

The localization of *Mbp* mRNA depends largely on its association with hnRNP A2 and the formation of RNA transport granules. We have previously shown that *Mbp* mRNA is translationally inhibited by sncRNA715 (Bauer et al., 2012). Small RNA-mediated post transcriptional gene regulation is mediated by the heterogeneous RISC complex which contains one of the four mammalian Ago proteins as a core component. The subfamilies of Ago (Ago1-4) and Piwi (HIWI1, HIWI2, HIWI3, HILI) proteins represent the Ago clade of the Ago protein family and interact with siRNAs and miRNAs (Pfaff and Meister, 2013). A functional RISC will consist of at least a small RNA and an Ago protein, but additional factors may be associated (Pratt and MacRae, 2009). Small RNA duplexes associate with Agos and form a preRISC structure which is transformed into the mature RISC after the RNA duplex has been processed and only a single guide RNA strand remains which is necessary to recognize target mRNAs by at least partial complementary sequence. In this study we investigated if Ago2 proteins are involved in these processes in oligodendrocytes and if they are part of the *Mbp* mRNA transport machinery. Ago2 has been shown to interact with the QKI-6 RNA binding protein in stress conditions in cytoplasmic granules and in turn QKI-6 colocalizes with *Mbp* mRNA in stress granules of U343 human glioblastoma cells, but an involvement of the miRISC was not suggested (Wang et al., 2010). So far a direct interaction of Ago2 and *Mbp* mRNA could not be demonstrated. In our study we analyzed a potential role of Ago2 during *Mbp* mRNA localization while the mRNA is repressed by sncRNA715 (Bauer et al., 2012). We could show that Ago1–4 is expressed by oligodendroglial cells and that the intracellular Ago2 distribution is granular and associates with hnRNP A2, *Mbp* mRNA, and sncRNA715 in oligodendrocytes. These findings strongly suggest an involvement of the RISC complex in the translational regulation of *Mbp* in RNA transport granules. It remains to be shown if this is the minimum functional complex or if other RISC components are associated in the *Mbp* mRNA localization pathway. It is likely that an interaction with GW182 proteins mediates downstream processes (Pfaff and Meister, 2013).

### Ago2 is a Downstream Target of Fyn Kinase

Translational activation of *Mbp* mRNA is mediated by oligodendroglial Fyn kinase responding to neuronal signals (White et al., 2008; Laursen et al., 2011; Wake et al., 2011). Fyn belongs to the Src family of non-receptor tyrosine kinases and in addition to translational regulation of *Mbp*, it controls cytoskeletal recruitment as well as morphological differentiation in oligodendrocytes (Kramer-Albers and White, 2011; White and Krämer-Albers, 2014). The lack of Fyn activity results in hypomyelination in the forebrain (Sperber et al., 2001) emphasizing its central role in oligodendrocyte signaling. Fyn phosphorylates the RNA granule proteins hnRNP A2 and hnRNP F and stimulates the translation of *Mbp* (White et al., 2008, 2011). It was postulated that these post-translational modifications result in a destabilization of the RNA granules and the release of *Mbp* mRNA so that it can be translated locally. It remains unclear, however, when and how the inhibitory action of sncRNA715 on *Mbp* is abolished. In addition to serine/threonine phosphorylation of Ago2, tyrosines Y393 and Y529 were identified by mass spectrometry to be phosphorylated in HEK293 cells. Interestingly, phosphorylation of Y529 which is located in the RNA binding pocket of the MID domain was shown to affect the association of small RNAs with Ago2 (Rudel et al., 2011). Therefore a model was suggested in which Ago proteins or their function in respect to small RNA-mediated translational control can be switched on or off by tyrosine kinases and phosphatases (Rudel et al., 2011). The phosphorylation of Y393 has been shown to decrease the interaction of Ago2 with Dicer and prevented miRNA loading to Ago2 (Shen et al., 2013; Yang et al., 2014). Hence both tyrosines seem to play a major role in regulation of Ago2 function. In the light of Fyn-mediated translational stimulation of *Mbp* mRNA and the inhibitory function of sncRNA715 in oligodendrocytes, we analyzed if Fyn phosphorylates Ago2. As active Fyn is located in lipid raft microdomains of the plasma membrane, this phosphorylation would occur at the site of MBP synthesis, at the axon glial contact site during active phases of myelination. We could indeed show a co-distribution of Fyn and Ago2 at these sites by co-immunostainings. Furthermore, the results we present here clearly identify Ago2 proteins as downstream targets of Fyn in oligodendrocytes. Future phospho-site analyses by mass spectrometry in response to Fyn manipulation will reveal the tyrosine residue which is phosphorylated by Fyn. It remains to be shown then how this post-translational modification will affect sncRNA715-mediated translational inhibition of *Mbp* mRNA. It may be the case that this phosphorylation will regulate the association of Ago2 with protein components of the RNA transport granules. Potentially the described phosphorylation of hnRNP A2 and hnRNP F as well as Ago2 will affect the association of these molecules with the RNA granule and mRNA cargo due to repulsive charges. Due to the involvement of Fyn kinase in different aspects of oligodendrocyte biology, we cannot rule out the possibility that Fyn-mediated phosphorylation of Ago2 affects cellular pathways independent of localized *Mbp* translation such as morphological differentiation via Rho GTPases or cytoskeletal reorganization during the onset of myelination (Kramer-Albers and White, 2011).

In summary we present data supporting the role of Ago2 proteins in the synthesis pathway of MBP and reveal Agos as downstream targets of Fyn kinase in oligodendrocytes. Future studies are required to analyze in more detail if and how Fyn mediated phosphorylation of Ago2 proteins affects the spatio-temporal control of MBP protein synthesis.

## Conflict of Interest Statement

The authors declare that the research was conducted in the absence of any commercial or financial relationships that could be construed as a potential conflict of interest.

## Supplementary Material

**FIGURE S1 | Ago1 and Ago2 are expressed in the murine *Nervus Opticus***. Optic nerves were extracted from mice postnatal day 1 (p1) or 10 (p10), lysed and analyzed via Western blotting for Ago2, Myelin basic protein (MBP), and GAPDH (loading control). Ago1 and Ago2 can be detected in the optic nerves of p1 and p10 mice. MBP signal in p10 indicates myelination ongoing *in vivo*.

**FIGURE S2 | Quantification for the colocalization of Ago2 and hnRNP A2 in mouse primary oligodendrocytes 5 days *in vitro* (5DIV)**. **(A)** Summary table of the Mander’s correlation coefficient (MCC) of 6 independent experiments (*n* = 6). The mean of M2 (fraction of green channel overlapping with red channel) and the SEM are indicated. **(B)** Summary table of MCC M1, Pearson’s correlation coefficient (PCC) and Cost’s randomized based colocalization test (Cost’s test). **(C)** Examples of quantified regions of interest in the most intense single confocal plane of the indicated experiment (n).

**FIGURE S3 | Quantification for the colocalization of Ago2 and Fyn kinase in mouse primary oligodendrocytes 3 days *in vitro* (3DIV)**. **(A)** Summary table of the Mander’s correlation coefficient (MCC) of 6 independent experiments (*n* = 6). The mean of M2 (fraction of green channel overlapping with red channel) and the SEM are indicated. **(B)** Summary table of MCC M1, Pearson’s correlation coefficient and Cost’s randomized based colocalization test (Cost’s test). **(C)** Examples of quantified regions of interest in the most intense single confocal plane of the indicated experiment (n).
