# Supplementary figures and images for: Oligodendroglial Argonaute protein Ago2 associates with molecules of the Mbp mRNA localization machinery and is a downstream target of Fyn kinase
Source: Front Cell Neurosci. 2015 Aug 25;9:328. doi: 10.3389/fncel.2015.00328 (PMC4548153; doi:10.3389/fncel.2015.00328)

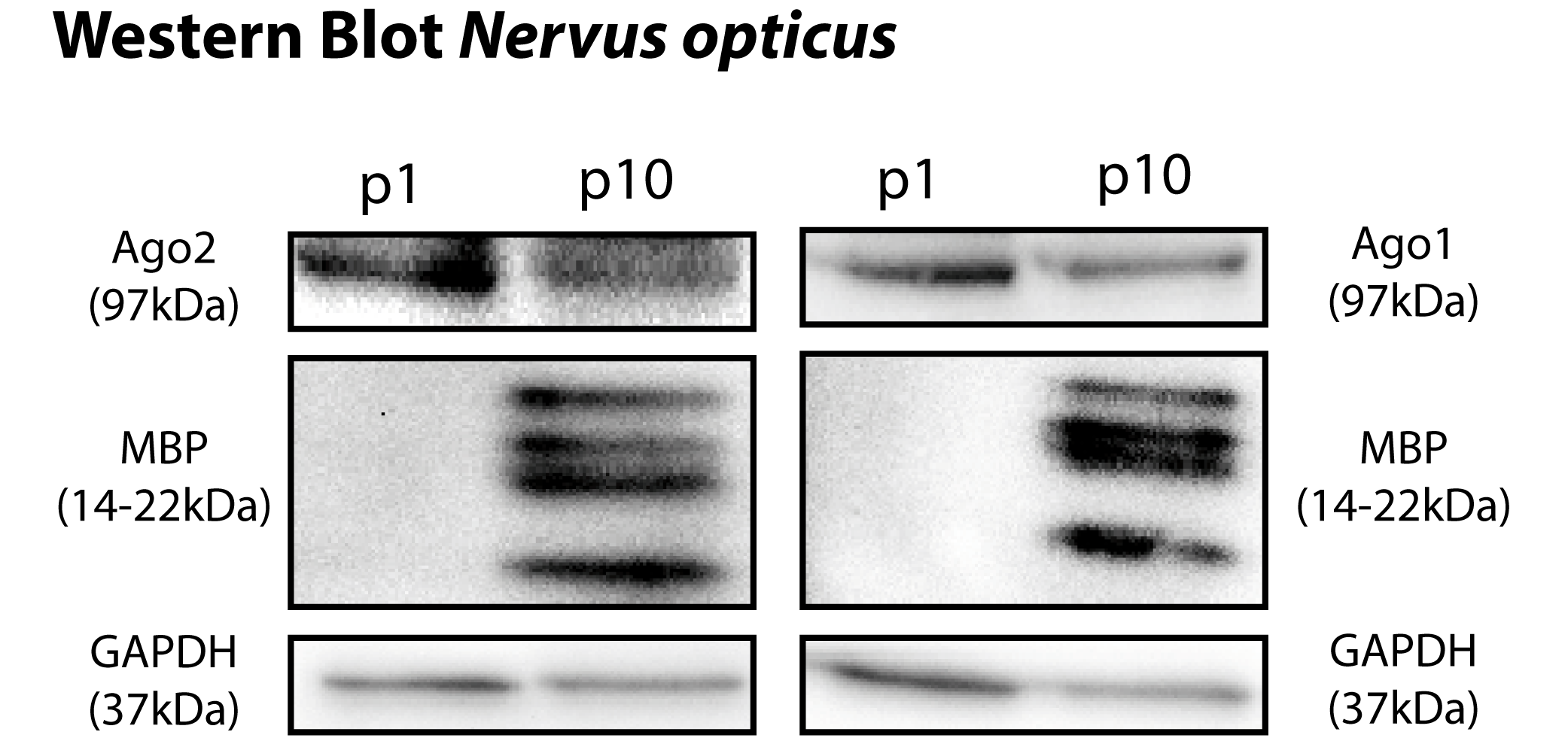

Supplement: Supplementary file 1 [file Image_1.TIF]

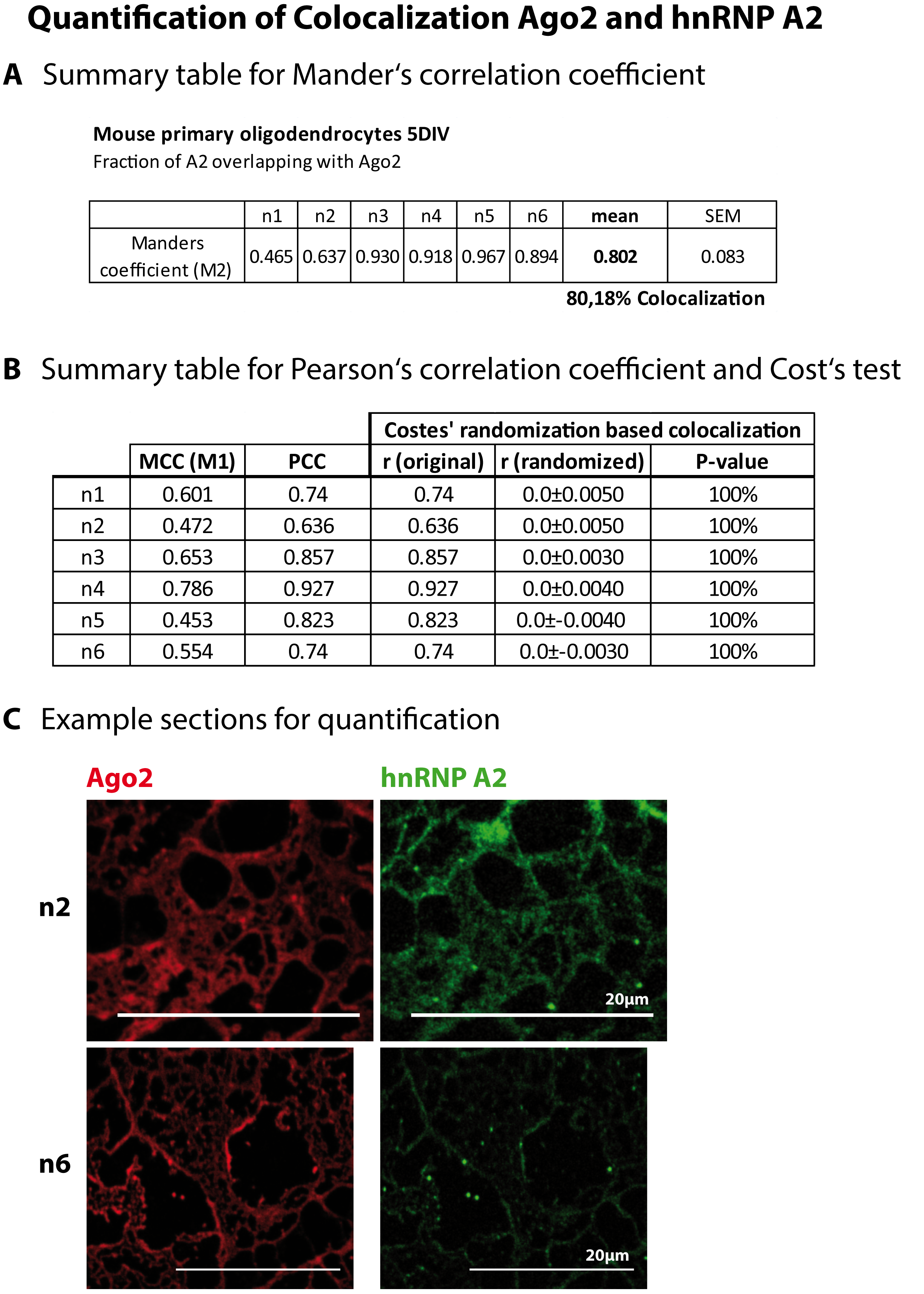

Supplement: Supplementary file 2 [file Image_2.TIF]

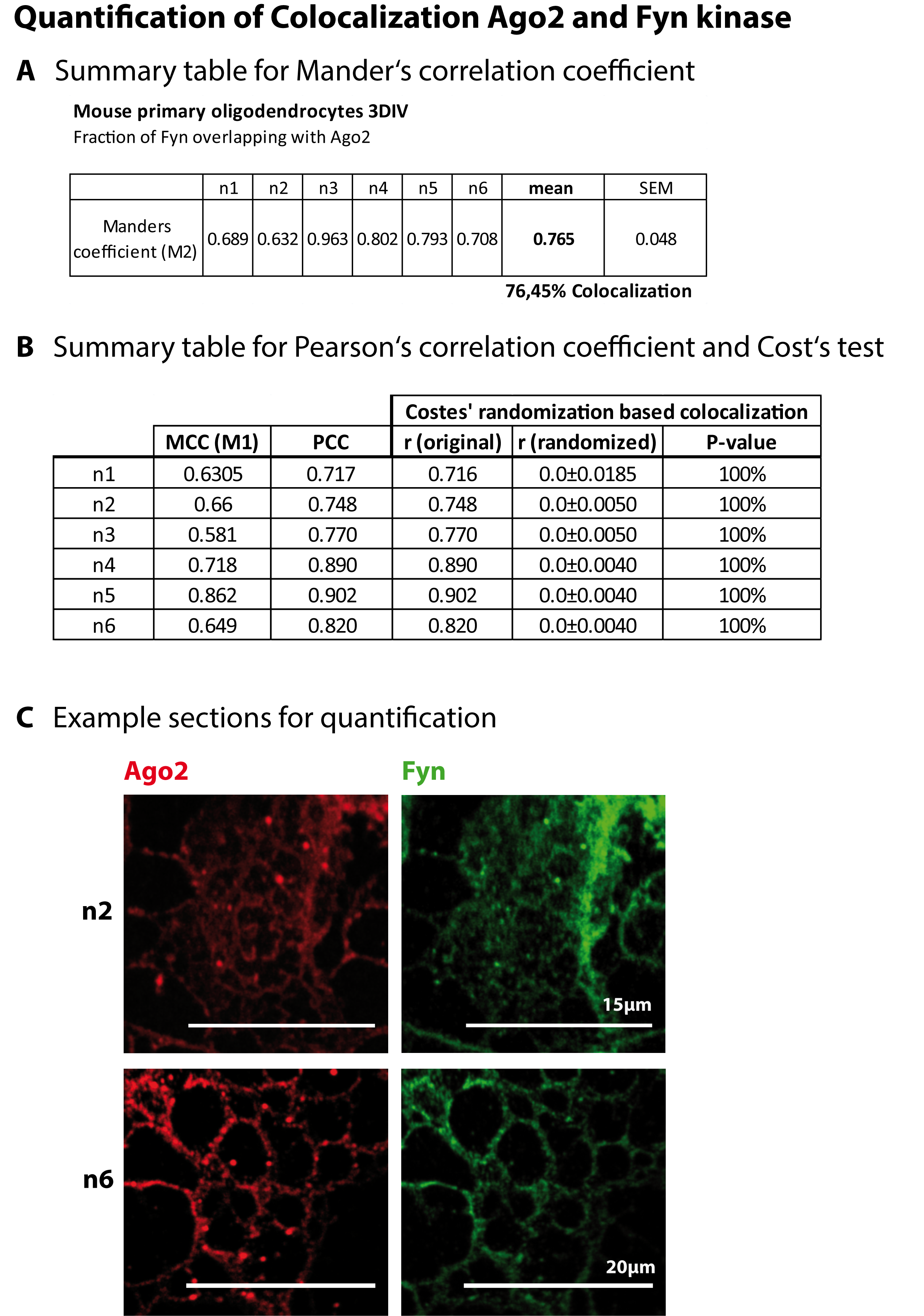

Supplement: Supplementary file 3 [file Image_3.TIF]
